# Quantum Mechanics Is Compatible with Counterfactual Definiteness

**DOI:** 10.3390/e25091356

**Published:** 2023-09-20

**Authors:** Janne V. Kujala, Ehtibar N. Dzhafarov

**Affiliations:** 1 Department of Mathematics and Statistics, University of Turku, FI-20014 Turun yliopisto, Finland; jvk@iki.fi; 2 Department of Psychological Sciences, Purdue University, West Lafayette, IN 47907, USA

**Keywords:** contextuality, counterfactual definiteness, strong consistent connectedness

## Abstract

Counterfactual definiteness (CFD) means that if some property is measured in some context, then the outcome of the measurement would have been the same had this property been measured in a different context. A context includes all other measurements made together with the one in question, and the spatiotemporal relations among them. The proviso for CFD is non-disturbance: any physical influence of the contexts on the property being measured is excluded by the laws of nature, so that no one measuring this property has a way of ascertaining its context. It is usually claimed that in quantum mechanics CFD does not hold, because if one assigns the same value to a property in all contexts it is measured in, one runs into a logical contradiction, or at least contravenes quantum theory and experimental evidence. We show that this claim is not substantiated if one takes into account that only one of the possible contexts can be a factual context, all other contexts being counterfactual. With this in mind, any system of random variables can be viewed as satisfying CFD. The concept of CFD is closely related to but distinct from that of noncontextuality, and it is the latter property that may or may not hold for a system, in particular being contravened by some quantum systems.

## 1. Introduction

A measurement has three characteristics. One is the measurement’s *content*: this is the question the measurement answers, or equivalently, the physical property whose value the measurement determines. The second characteristic of a measurement is its *context*: this includes other measurements made together with this one, and the spatiotemporal relations among them. The “togetherness” of two measurements means that there is an empirical rule by which the outcomes of these measurements are paired. The third characteristic of a measurement is the *probability distribution* of its values. More precisely, all the measurements made in the same context possess a *joint distribution* which determines the distribution of any given measurement.

*Counterfactual definiteness* (CFD) of a measurement is its compliance with the following counterfactual statement: had the measurement with the same content been made in another context, its outcome would have been the same. We will argue that under an assumption commonly accepted in quantum mechanics, CFD is always satisfied.

We begin by illustrating the terms and notions mentioned above (and to be formally defined in Section 2) using a toy example. It is based on the parable of the seer of Nineveh that was introduced by Ernst Specker [1] and subsequently used by others as a simple example of contextuality [2]. Omitting the colorful story line, the seer of Nineveh had three boxes obeying the following Magic Box Rules: (MBR1) only two of them could be opened at any given time; (MBR2) regardless of which two boxes were opened, one and only one of them contained a gem, and (MBR3) the gem could be contained in either of the two with equal probabilities. We can take these Magic Box Rules as an analogue of the laws of quantum mechanics. A formal representation of this situation is by the following *system of random variables*:

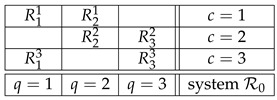
(1)It represents six measurements $R_{q}^{c}$ having three *contents q* and made pairwise in three *contexts c*. The content *q* of $R_{q}^{c}$ can be thought of as the question “does the box *q* contain a gem?” (q=1,2,3). This question is answered Yes or No, which are the possible values of $R_{q}^{c}$. Equivalently, we can view *q* as the property “the contents of the box *q*” (q=1,2,3), in which case the possible values of $R_{q}^{c}$ are “gem” and “no gem.” Irrespective, we will denote the values of $R_{q}^{c}$ as +1 and −1. The context *c* indicates which two boxes have been opened. All other conditions under which the measurement are made (e.g., the shape of the boxes) are the same in the three contexts, so we do not list them in the definition of a context. Contexts are always mutually exclusive, by definition: random variables measured in different conditions never co-occur, there is no empirical rule for pairing the values of, say, $R_{1}^{1}$ and $R_{3}^{2}$.

The rules MBR2 and MBR3 say that in each of the three contexts, the two measurements $R_{q}^{c}$ and $R_{q^{\prime }}^{c}$ made in it have the joint distribution

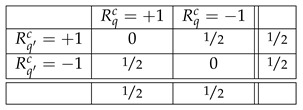
(2)The individual distribution of each $R_{q}^{c}$, by MBR3, is one and the same for all random variables: +1 and −1 with equal probabilities. In particular, it is the same for any two measurements answering the same question in different contexts, such as $R_{2}^{1}$ and $R_{2}^{2}$. If one repeatedly observes openings of the box q=2, and sees no other boxes, one has no way of determining in which context the box is being opened, in the one with the box q=1 or with the box q=3. This is a special case of an assumption we are going to make throughout this paper: in the quantum mechanical literature it is known under variety of names, such as *non-signaling* or *non-disturbance* [3,4,5]. In Section 2, we will define a strong version of this notion following Abramsky and Brandenburger [6].

### 1.1. Noncontextual Representation of Variables

A standard way of introducing the notion of contextuality, applying it to our example, is to ask:

Q0: is it possible to treat all random variables in the system as if any two variables with the same content were identical?

For instance, $R_{1}^{1}$ and $R_{1}^{3}$ have the same content, both answer the question q=1: “Does the box #1 contain a gem?”. The other boxes opened together with the box #1 (i.e., the box #2 in context c=1 or the box #3 in context c=3) in no way affect the distribution of the possible answers to the question q=1. Someone who observes the box #1 repeatedly, without seeing the other boxes, has no way of determining the context of the box #1 when it is opened. Therefore it seems it should be possible to simply view $R_{1}^{1}$ and $R_{1}^{3}$ as one and the same variable. Analogous reasoning applies to other pairs of measurements sharing a content, $\left(R_{2}^{1},R_{2}^{2}\right)$ and $\left(R_{3}^{2},R_{3}^{3}\right)$.

However, it is easy to see that this *noncontextual representation* of the variables in our example is not possible. Let us begin by renaming $R_{1}^{1}$ into *X*, and then proceed by identifying other random variables following the Magic Box Rules and noncontextual representation. The first step will yield

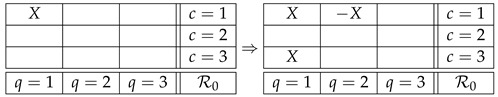
(3)
where the −X in the first row follows MBR2, and the second *X* in the first column follows noncontextual representability. Proceeding in this manner, we obtain

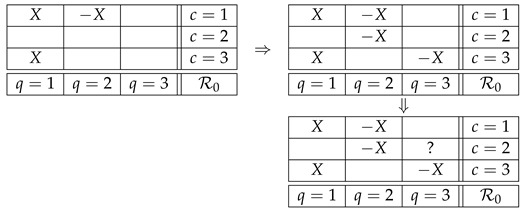
(4)
and we see that the cell with “?” cannot be filled, as it should be −X to maintain noncontextual representation in the third column but it should be *X* to follow MBR2 in the second row. The conclusion is that no noncontextual representation of the random variables in our system exists. When this happens, a system is said to be *contextual* (otherwise it is *noncontextual*).

### 1.2. Counterfactual Definiteness

One can, however, approach our system in a different way. Given that a box was opened in some context (that we will call the *factual context*), one can ask: had this box been opened in another context (called *counterfactual*), would the outcome have been the same? The term “outcome” has two meanings: “random variable” and “value of a random variable.” In the present context, however, the two are interchangeable, and the counterfactual question can also be formulated thus: had this box been opened in another context, would the counterfactual variable $R^{\prime }$ have been representable by the same random variable as the factual one, *R*? The reason for this is that we can think of the counterfactual question about values of the variables being asked repeatedly, and $R^{\prime }$ and *R* can always have the same value if and only if $R^{\prime }=R$.

In the contextuality literature the counterfactual question above is considered to be logically equivalent to Q0 [7,8,9,10,11,12]. However, a detailed analysis shows this is not the case. Using our example (1), consider the situation when the factual context is c=2, i.e., we observe the values of the *factual variables* $R_{2}^{2}$ and $R_{3}^{2}$. One can then ask two counterfactual questions:

Q1: if instead of $R_{2}^{2}$ one had recorded $R_{2}^{1}$ (the same box in context c=1), would $R_{2}^{1}$ have been the same as $R_{2}^{2}$?
Q2: if instead of $R_{3}^{2}$ one had recorded $R_{3}^{3}$ (the same box in context c=3), would $R_{3}^{3}$ have been the same as $R_{3}^{2}$?

It is easy to see that in our example the answer to both questions is affirmative, in the sense that there is nothing in the Magic Box Rules that would prevent one from considering a counterfactual variable identical to the corresponding factual one. Denoting $R_{2}^{2}$ by *X*, we have, for Q1,

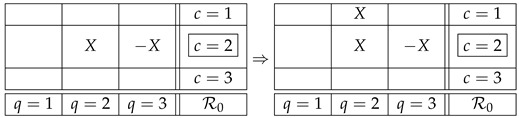
(5)Moreover, this representation can never come into a conflict with other variables in the same counterfactual context:
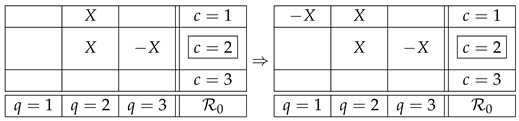
(6)We can repeat the same reasoning for Q2:
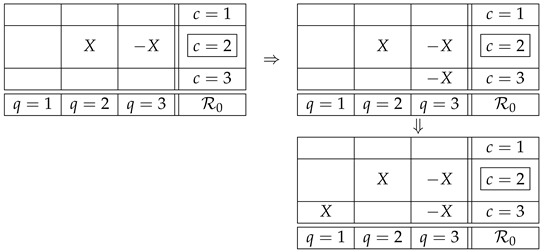
(7)We have here a special case of the general theorem proved in the next section: it says that if a system satisfies the no-disturbance condition, then for any factual context and any counterfactual one, the variables in the latter can be chosen so that the same-content variables in the two contexts are identical. That is, any system with no disturbance has the property of CFD.

Returning to our example, however, we have a natural question to ask: What if the questions Q1 and Q2 are answered together? Would we not run into a contradiction then? What we have is

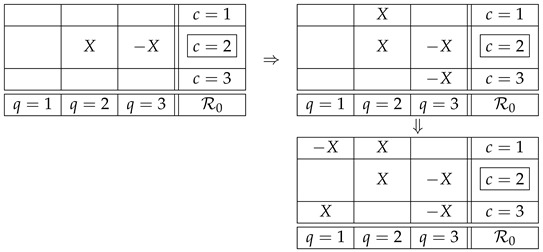
(8)And it seems that we indeed have run into a contradiction, because in the first column the variables are not the same. However, one can notice this only if one compares two counterfactual contexts to each other with the purpose of determining if they comply with noncontextual representability. In other words, one notices this contradiction if the question one answers is Q0 rather than Q1 and Q2.

We already know that the system is contextual, i.e., Q0 is answered in the negative. What we should be interested in now is whether a contradiction occurs if we deal only with the counterfactual questions, without explicitly involving noncontextual representability. It is clear, however, that one cannot formulate purely counterfactual questions to compare two counterfactual contexts without making one of them factual. It is logically impossible.

The principal difference between noncontextual representability and CFD is that the latter puts the system into a frame of reference formed by the choice of a factual context. Changing the factual context changes the frame of reference. In this picture, noncontextual representability can be viewed as the possibility of reconciling all different frames of reference. However, this is an additional and different question—about contextuality. A system may be contextual or noncontextual, but CFD is satisfied always.

## 2. Formal Treatment of Contextuality and Counterfactual Definiteness

### 2.1. Basic Notions

We begin by defining the notions discussed in the previous section in a more rigorous way. The terminology and notation we use are those developed in the Contextuality-by-Default (CbD) approach to contextuality [13,14]. Although we have presented an example of a system of random variables in the opening section, it was a very specially constructed system (uniform dichotomous distributions and perfect anticorrelations in each context). We think therefore it is useful to provide additional illustrations using an example of a more generic variety:
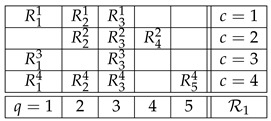
(9)In parallel, we will also use for illustrations a realisitic example, the system of random variables for which John Bell and others derived the celebrated inequalities bearing his name [15,16,17]:
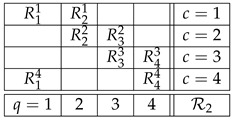
(10)This system describes the EPR/Bohm experiment [18] with two entangled spin-½ particles whose spins are measured by two respective spacelike-separated experimenters traditionally designated as Alice and Bob. The contents q=1 and q=3 designate the settings (axes) that may be chosen by Alice, and Bob’s settings are designated by q=2 and q=4. Mathematically, system $R_{2}$ is less interesting than system $R_{1}$ (the former being essentially of the same structure as our opening toy example). However, $R_{2}$ has the distinction of having dominated the discussions related to contextuality (in the form of nonlocality) in the literature on the foundations of quantum mechanics.

In complete generality, a system of random variables is an indexed set

(11) $$R=\left\{R_{q}^{c}:q\in Q,\;c\in C,\;q≺c\right\},$$

where *Q* and *C* are sets of contents and contexts, respectively, and q≺c indicates that *q* is measured in *c*, with the outcome $R_{q}^{c}$ a random variable. In each context the variables possess a joint distribution, whereas there are no joint distributions across the contexts.

The notion defined next uses a CbD term for what is usually referred to as non-disturbance or non-signaling, understood in the strong sense of the term formalized by Abramsky and Brandenburger [6].

**Definition** **1.** *A system R in (11) is* strongly consistently connected *(s.c.c.) if, for any $Q^{\prime }\subseteq Q$ and any c∈C such that q≺c for all $q\in Q^{\prime }$, the joint probability distribution of $\left\{R_{q}^{c}:q\in Q^{\prime }\right\}$ only depends on $Q^{\prime }$ [13].*

We will assume that both our example systems, (9) and (10), are s.c.c. (for system $R_{2}$ this follows from the spacelike separation of Alice and Bob). In $R_{1}$, choosing $Q^{\prime }$ as $\left\{2,3\right\}$, we have the identically distributed pairs $\left\{R_{2}^{1},R_{3}^{1}\right\}$, $\left\{R_{2}^{2},R_{3}^{2}\right\}$, and $\left\{R_{2}^{4},R_{3}^{4}\right\}$. In $R_{2}$, choosing again $Q^{\prime }$ as $\left\{2,3\right\}$, we have identically distributed $R_{3}^{2}$ and $R_{3}^{3}$.

**Definition** **2.** *An s.c.c.-system R in (11) is* noncontextual *if there is a jointly distributed set of random variables $S=\left\{S_{q}:q\in Q\right\}$, such that, for any $Q^{\prime }\subseteq Q$ and any c∈C with q≺c for all $q\in Q^{\prime }$, the distribution of $\left\{R_{q}^{c}:q\in Q^{\prime }\right\}$ is the same as the distribution of $\left\{S_{q}:q\in Q^{\prime }\right\}$. The set of variables S is referred to as a* reduced coupling *of R [19].*

Applying this definition to system $R_{1}$ in (9), it is noncontextual if one can find five jointly distributed variables $\left\{S_{1},\ldots ,S_{5}\right\}$ such that

(12) $$\left\{R_{1}^{1},R_{2}^{1},R_{3}^{1}\right\}\overset{d}{=}\left\{S_{1},S_{2},S_{3}\right\},\;\left\{R_{2}^{2},R_{3}^{2},R_{4}^{2}\right\}\overset{d}{=}\left\{S_{2},S_{3},S_{4}\right\},\;\mathrm{etc}.,$$

with $\overset{d}{=}$ standing for equality of distributions. For system $R_{2}$, noncontextuality means the existence of four jointly distributed variables $\left\{S_{1}^{\prime },\ldots ,S_{4}^{\prime }\right\}$ such that

(13) $$\left\{R_{1}^{1},R_{2}^{1}\right\}\overset{d}{=}\left\{S_{1},S_{2}\right\},\;\left\{R_{2}^{2},R_{3}^{2}\right\}\overset{d}{=}\left\{S_{2},S_{3}\right\},\;\mathrm{etc}.$$

It is well known that this condition is equivalent to

(14) $$\max\left(\pm \langle R_{1}^{1}R_{2}^{1}\rangle \pm \langle R_{2}^{2}R_{3}^{2}\rangle \pm \langle R_{3}^{3}R_{4}^{3}\rangle \pm \langle R_{4}^{4}R_{1}^{4}\rangle \right)\leq 2,$$

where the maximum is taken over the eight choices of the ± signs with odd number of minus signs. (This is the CHSH inequality [16], with all variables’ values assumed to be ±1.)

The next notion formalizes the intuitive meaning of the following statement: The essence of noncontextuality for s.c.c. systems is that all content-sharing random variables can be treated *as if they were* one and the same variable.

**Definition** **3.** *An* identically connected (i.c.) coupling *of a noncontextual s.c.c. system R in (11) is an indexed set*

(15) $$S^{*}=\left\{S_{q}^{c}:S_{q}^{c}=S_{q},\;q\in Q,\;c\in C,\;q≺c\right\},$$

*where $S_{q}$ is an element of the reduced coupling of the system.*

Thus, in our two examples, (9) and (10), if the systems $R_{1}$ and $R_{2}$ are noncontextual, then their variables can be viewed *as if they were*, respectively,

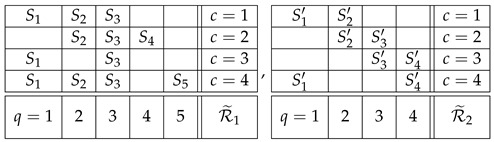
(16)These systems of variables are i.c. couplings of, respectively, $R_{1}$ and $R_{2}$.

### 2.2. Factual-Counterfactual Subsystems

Any context in a system can be chosen and designated as a *factual context*. The variables recorded in this context are called *factual variables*. All other contexts and the variables they contain are referred to as *counterfactual*.

**Definition** **4.** *Having chosen a factual context, $c=c_{0}$, a subsystem $R⟦c_{0}⟧$ of the system R in (11) is called a* factual-counterfactual *(F-CF) subsystem (with respect to $c_{0}$) if it consists of all variables that share their contents with the factual variables.*

This subsystem, of course, includes the factual variables themselves. Presented explicitly, the F-CF subsystem of (11) with respect to $c_{0}$ is

(17) $$R⟦c_{0}⟧=\left\{R_{q}^{c}:q\in Q,\;c\in C,\;q≺c,c_{0}\right\},$$

where $q≺c,c_{0}$ means q≺c and $q≺c_{0}$. Thus, in the examples (9) and (10), if we choose c=2 as a factual context in each of them, then the respective F-CF subsystem will be

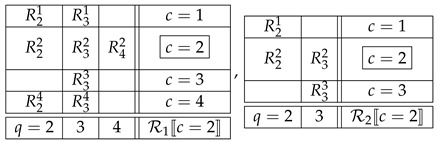
(18)

### 2.3. Counterfactual Definiteness

We are ready now to rigorously formulate CFD in terms of the noncontextuality of the F-CF subsystems of a system.

**Definition** **5.** *An s.c.c. system is said to have the property of* counterfactual definiteness *(CFD) if all its F-CF subsystems are noncontextual.*

The justification of this definition lies in the intuition formalized by the notion of an i.c. coupling. If the F-CF subsystems in (18) are noncontextual, then their i.c. couplings are, respectively,

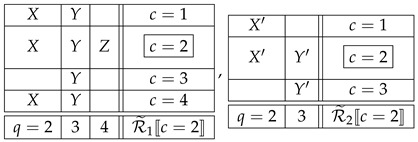
(19)
where $\left\{X,Y,Z\right\}$ and $\left\{X^{\prime },Y^{\prime }\right\}$ are reduced couplings of the respective F-CF subsystems. It is *as if* all counterfactual variables were the same as the corresponding factual ones. Moreover, in each of the counterfactual contexts these representations of the two F-CF subsystems are compatible with the overall joint distributions in this context: 
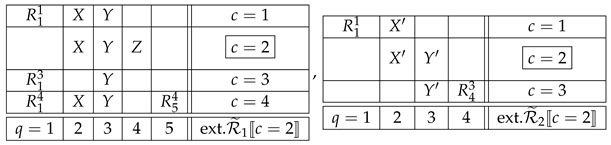
(20)
where “ext.” abbreviates “extended.” This is a representation (coupling) of the system (9) with a factual context c=2, in which any counterfactual variable sharing a content with a factual one is identical with the latter. This is the intuitive meaning of CFD.

### 2.4. Universality of Counterfactual Definiteness

It is easy to see by inspecting (19) that the noncontextuality of this F-CF subsystem did not have to be assumed: e.g., if $R_{1}$ in (9) is s.c.c., the noncontextuality of $R_{1}⟦c=2⟧$ in (18) clearly holds by choosing the reduced coupling $\left\{X,Y,Z\right\}$ as $\left\{R_{2}^{2},R_{3}^{2},R_{4}^{2}\right\}$. The same holds for other three F-CF subsystems of (9), as well as for the four F-CF subsystems of (9), which means that the systems $R_{1}$ and $R_{2}$ both satisfiy CFD.

**Theorem** **1.** *Any s.c.c. system satisfies CFD.*

**Proof.**  Let R in (11) be a s.c.c. system. We need to show that any of its F-CF subsystems is noncontextual. Let $c=c_{0}$ to be a factual context. Then the jointly distributed set of variables

(21) $$R^{c_{0}}=\left\{R_{q}^{c_{0}}:q\in Q,\;q≺c_{0}\right\}$$

is a reduced coupling of $R⟦c_{0}⟧$. Indeed, for any c∈C,

(22) $$R^{c}=\left\{R_{q}^{c}:q\in Q,\;q≺c,c_{0}\right\}\overset{d}{=}\left\{R_{q}^{c_{0}}:q\in Q,\;q≺c_{0},c\right\},$$

because the system is s.c.c. □

To emphasize once again the underlying intuition, it follows from the theorem that any F-CF subsystem has an i.c. coupling

(23) $$S^{*}⟦c_{0}⟧=\left\{S_{q}^{c}:S_{q}^{c}=R_{q}^{c_{0}},\;q\in Q,\;c\in C,\;q≺c,c_{0}\right\}.$$

In other words, for any counterfactual context $c\neq c_{0}$ and any $q≺c_{0},c$, the counterfactual variable $R_{q}^{c}$ can be treated *as if it were* $R_{q}^{c_{0}}$.

## 3. Concluding Remarks and Possible Generalizations

### 3.1. Counterfactual Definiteness and Noncontextuality

We have shown that compliance with CFD and noncontextuality of a system of random variables are related but different concepts: CFD is always satisfied as a consequence of non-disturbance (s.c.c.) property, irrespective of whether the system is contextual. The mathematical reason for this is that a counterfactual question creates a single frame of reference, a factual context, which, together with the variables in the counterfactual contexts to which the question pertains, forms a noncontextual subsystem. The overall (non)contextuality is a property without such a frame of reference, or one in which all different frames of reference are reconciled. However, to achieve such a reconciliation, or to establish that it is not possible, one has to use conceptual means that cannot be presented in the form of counterfactual questions.

Interestingly, David Mermin, in his well-known paper [20], makes a distinction between *Strong Baseball Principle*, corresponding to CFD, and *Very Strong Baseball Principle*, corresponding to overall noncontextuality. Except for terminological and expository differences, the Strong Baseball Principle is introduced as in our example (8), by choosing a factual context and creating two counterfactual ones following CFD. Then Mermin compares the two counterfactual contexts to each other, and says: “This last application of the Strong Baseball Principle, by comparing hypothetical cases, has a different character than the first two, which compare a hypothetical case with the real one, and here it might more accurately be termed the Very Strong Baseball Principle.” Mermin proceeds, however, by arguing that the latter should not be treated separately from the Strong Baseball Principle, the argument being that comparing counterfactual contexts is as “reasonable” and “permissible” as comparing them with a factual context. One of the authors of the present paper maintained the same position in Ref. [12], using similar arguments. Our position here is that being equally reasonable and permissible, CFD and noncontextuality are logically distinct principles, of which only the latter may fail to hold in s.c.c. systems.

We find a similar situation in a 1981 paper by John Clauser and Abner Shimony [9]. They analyze Henry Stapp’s [7] approach to the derivation of Bell’s inequalities, according to which they follow from certain four equations. These equations can be interpreted as stating that CFD holds for the four F-CF subsystems of the EPR/Bohm system, i.e., $R_{2}$ in our example (10). Thus, Stapp’s position is that whenever $R_{2}$ is contextual, it must contravene CFD. Clauser and Shimony point out that Stapp’s equations only apply to the pairs of contexts (F-CF pairs, in our terminology), and that “Stapp has not given a reason for demanding the existence of a quadruple of possible worlds which mesh together [these four pairs]”. However, they seem to accept Stapp’s response to this objection, which we do not present here as, in our view, it misses the point. Clauser and Shimony’s objection is accepted as valid by Bernard d’Espagnat [21], but his position, in contrast to ours, seems to be skeptical of the meaningfulness of CFD altogether.

For completeness, we should mention that Robert Griffiths [22,23] also argues that CFD is always satisfied in quantum mechanics. His argumentation, however, is very different from ours. Moreover, unlike in our paper, Griffiths considers CFD to be completely unrelated to noncontextuality.

### 3.2. Systems with Signaling/Disturbance

CbD provides a generalization of the notion of (non)contextuality to systems that are not necessarily s.c.c. In fact, the distributions of the random variables in the system can be arbitrary. It is interesting to see if CFD generalizes similarly, and if so, what the relations are between generalized (non)contextuality and generalized CFD.

**Definition** **6.** *A system R in (11) is considered noncontextual in CbD if it has a* multimaximally connected *coupling, defined as an indexed set of jointly distributed variables*

(24) $$S^{*}=\left\{S_{q}^{c}:q\in Q,\;c\in C,\;q≺c\right\}$$

*such that, (1) for any $Q^{\prime }\subseteq Q$ and any c∈C with q≺c for all $q\in Q^{\prime }$, the distribution of $\left\{R_{q}^{c}:q\in Q^{\prime }\right\}$ is the same as the distribution of $\left\{S_{q}:q\in Q^{\prime }\right\}$; and (2) the probability of $S_{q}^{c}=S_{q}^{c^{\prime }}$, for any $q≺c,c^{\prime }$, is maximal possible.*

For s.c.c. systems, this definition specializes to Definition 2, with the maximal probability of $S_{q}^{c}=S_{q}^{c^{\prime }}$ being 1 (because of which they both can be renamed into $S_{q}$).

One can now apply Definition 5 to an arbitrary system R:

**Definition** **7.** *A system is said to satisfy generalized CFD (gCFD), if all of its F-CF subsystems are noncontextual.*

The intuitive meaning of gCFD is that the outcome of a counterfactual measurement, had it been made, would have been the same as the factual one with the highest possible probability (given the distributions of the two measurements).

We now have no analogue of Theorem 1. Based on the general properties of (non)contextuality, one can only say that (A) if a system is noncontextual then so are all of its F-CF subsystems (i.e., the system satisfies gCFD); and (B) if a system does not satisfy gCFD, then it is contextual. Moreover, all s.c.c. systems provide evidence that compliance with gCFD (in the form of CFD) does not imply noncontextuality. All *cyclic systems* [24], even if not s.c.c. [25], demonstrate the same. For instance, the system $R_{2}$ in our example (10) is contextual whenever

(25) $$\begin{array}{c} \max\left(\pm \langle R_{1}^{1}R_{2}^{1}\rangle \pm \langle R_{2}^{2}R_{3}^{2}\rangle \pm \langle R_{3}^{3}R_{4}^{3}\rangle \pm \langle R_{4}^{4}R_{1}^{4}\rangle \right)>2+\left|\langle R_{1}^{1}\rangle -\langle R_{1}^{4}\rangle \right|\\ +\left|\langle R_{2}^{2}\rangle -\langle R_{2}^{1}\rangle \right|+\left|\langle R_{3}^{3}\rangle -\langle R_{3}^{2}\rangle \right|+\left|\langle R_{4}^{4}\rangle -\langle R_{4}^{3}\rangle \right|, \end{array}$$

with the same meaning of the terms as in (14), which is a special case of (25). At the same time, every F-CF subsystem of this system is trivially noncontextual (because single-variable rows cannot contribute to contextuality): 
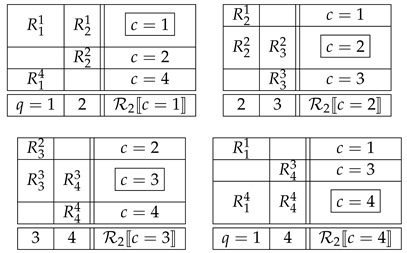
(26)More work is needed to find out if gCFD and more generally F-CF systems may productively complement the notion of (non)contextuality in the theory of systems of random variables.

## Data Availability

No data is associated with the paper.

## Abbreviations

The following abbreviations are used in this manuscript:

CbD	Contextuality-by-Default (approach to contextuality)
CFD	counterfactual definiteness
F-CF	factual-counterfactual (subsystem of random variables)
gCFD	generalized counterfactual definiteness
i.c.	identically connected (coupling)
MBR	“magic box rule”
s.c.c.	strongly consistently connected (without disturbance, non-signaling)
